# Extracellular microvesicles from patients with Rheumatoid arthritis promote dendritic cell activation *in vitro*

**DOI:** 10.3389/fimmu.2025.1532114

**Published:** 2025-03-05

**Authors:** Brigitta Buttari, Serena Recalchi, Gloria Riitano, Antonella Capozzi, Federica Maria Ucci, Valeria Manganelli, Federica Fratini, Elisabetta Profumo, Tina Garofalo, Cristiano Alessandri, Roberta Misasi, Fabrizio Conti, Agostina Longo, Maurizio Sorice

**Affiliations:** ^1^ Department of Cardiovascular and Endocrine-metabolic Diseases, and Aging, Istituto Superiore di Sanità, Rome, Italy; ^2^ Department of Experimental Medicine, “Sapienza” University of Rome, Rome, Italy; ^3^ Rheumatology Unit, Department of Clinical Internal, Anesthesiological and Cardiovascular Sciences, “Sapienza” University of Rome, Rome, Italy; ^4^ Proteomics Core Facility, Istituto Superiore di Sanità (ISS), Rome, Italy

**Keywords:** rheumatoid arthritis, dendritic cells, extracellular microvesicles, post-translational modifications, cell activation

## Abstract

**Introduction:**

Rheumatoid Arthritis (RA) is a systemic autoimmune disease characterized by chronic synovial inflammation affecting diarthrodial joints, with cartilage destruction and bone erosion. Environmental inflammatory stimuli can induce maturation of dendritic cells (DCs), which promote differentiation and activation of effector T lymphocytes. We previously highlighted the role of extracellular microvesicles (EMVs) in pathogenesis by carrying antigens that trigger autoantibody production. In this investigation we verified whether EMVs may activate immature monocyte-derived DCs, inducing phenotypic and functional characteristics of mature DCs.

**Methods:**

EMVs were obtained from 7 RA patients naïve to biological disease-modifying anti-rheumatic drugs (DMARDs) and tested for their capability to activate DCs from healthy donors.

**Results:**

We preliminary confirmed by western blot that carbamylated and citrullinated proteins are present in EMVs from RA patients. Moreover, surface marker phenotyping indicated that EMV treated-DCs exhibit increased expression of CD83 and CD86, as well as of CD83+ HLA-DR+ CD80+ CD86+ cells, indicating that the DCs are in a mature state. Furthermore, biochemical data demonstrated that EMVs from plasma of RA patients induce MAPK and NF-κB activation in DCs. EMVs from the plasma of RA patients were also able to stimulate DCs to produce IL-12, IL-1β and IL-10, inducing a proinflammatory phenotype.

**Conclusions:**

These findings demonstrate that EMVs from RA patients promote DC activation *in vitro*, suggesting a potential mechanism by which RA microenvironment perpetuates inflammation through the modulation of DC function. These knowledges provide new insight in the role of EMVs in the pathogenesis of RA and their potential role as therapeutic targets.

## 1 Introduction

Rheumatoid Arthritis (RA) is a systemic autoimmune disease characterized by chronic synovial inflammation, primarily affecting diarthrodial joints, leading to cartilage destruction and bone erosion. The etiology of RA is heterogeneous, involving intricate interactions among genetic predisposition, environmental factors, and dysregulated immune responses, affecting approximately 0.5-1% of the global population (1–3).

Environmental inflammatory stimuli can induce mature major histocompatibility complex (MHC) class II+ dendritic cells (DCs), which prime autoantigen-specific CD4+ T cells, including follicular helper T (Th) cells (4). Thus, DCs contribute to chronic RA inflammation maintenance by promoting the differentiation and activation of effector T cells, which produce pro-inflammatory cytokines that drive synovial inflammation (5). In addition, they cause a breach of self-tolerance leading to the production of autoantibodies against several proteins’ post-translational modifications (PTMs), mainly anti-citrullinated protein/peptide antibodies (ACPA) and anti-carbamylated proteins (anti-CarP) (6–8). Alterations of PTMs, which affect protein charge, structure, localization, interaction and function, can affect many cellular processes. Specifically, aberrant PTMs of proteins involved in DC maturation, antigen presentation and cytokine production have been implicated in RA pathogenesis (9). Indeed, PTMs involve covalent additions of functional groups to proteins, playing a vital role in maintaining their structure, function and stability. Moreover, emerging evidence shows that crucial regulatory mechanisms modulating protein function and activity contribute to RA pathogenesis by influencing the function of key proteins involved in immune regulation and inflammation (10, 11).

Two particular PTMs, citrullination and carbamylation, are crucial in RA pathogenesis (12, 13) upon activation of different molecular pathways, including autophagy (14, 15). Protein citrullination is an irreversible enzymatic PTM that converts peptidyl-arginine to peptidyl-citrulline, catalyzed by the peptidyl arginine deiminase (PAD) enzyme family. This modification induces electrostatic and conformational changes in the modified protein, affecting its function by altering binding sites, protein-protein interactions, and susceptibility to degradation. Citrullinated peptides are present in RA, and ACPA are key serological markers for RA diagnosis, with significant specificity and sensitivity. Principal citrullinated proteins in RA patients include alpha-enolase-1, vimentin, and type II collagen (16).

Carbamylation is a nonenzymatic reaction involving the binding of a “carbamoyl” group to the free functional groups of proteins, peptides, and free amino acids. This process is mediated by isocyanic acid, often produced through the enzymatic action of myeloperoxidase on thiocyanate in the presence of hydrogen peroxide. Anti-CarP antibodies, generated in response to carbamylated proteins, are involved in RA, particularly in patients negative for ACPA (17, 18).

We have demonstrated that post-translational modified proteins, especially carbamylated and citrullinated proteins are present on the surface of circulating extracellular microvesicles (EMVs) (19, 20). EMVs consist of a lipid bilayer enclosing proteins, peptides, lipids and genetic materials, physiologically released from cells into the extracellular space by the outward budding and pinching of the plasma membrane. Increased EMVs in various biological fluids of RA patients have been correlated with disease activity (21). We previously demonstrated a higher concentration of EMVs in the plasma of RA patients compared to healthy donors, highlighting their role in pathogenesis by carrying antigens that trigger autoantibody production (19).

A previous study showed that EMVs containing antigenic proteins, from an infectious agent, are able to interact with DCs, inducing their maturation and modulating the expression of surface molecules such as CD40, CD80, MHC I, and MHC II (22).

Although the pathogenetic role of DCs in the initiation and perpetuation of the autoimmune responses in RA has been extensively investigated, important insights regarding the signaling pathways that operate in normal and diseased conditions initiated by EMVs are lacking.

Starting from the evidence showing elevated levels of EMVs in the plasma of RA patients, as well as their association with antigens involved in autoantibody production, our objective in this study was to explore the possible interaction between EMVs derived from RA patients naïve to biological disease-modifying anti-rheumatic drugs (DMARDs) and DCs. Using immunochemical and cytofluorimetric analysis, we investigated whether PTMs could confer to EMVs the ability to activate immature monocyte-derived DCs from healthy human donors, inducing phenotypic and functional characteristics typical of mature DCs.

## 2 Methods

### 2.1 Patients

We enrolled 7 consecutive RA patients, naïve to biological therapy, satisfying the 2010 ACR RA criteria (23), from the Arthritis Center at the Sapienza University of Rome, and as the control group, 7 healthy donors (HDs), matched for age and sex with patients. The study protocol was approved by the Ethics Committee of Sapienza University of Rome, and informed written consent was obtained from all participants before enrolment. The clinical and demographic characteristics of RA patients are shown in **Table 1**.

**Table 1:** Clinical parameters of RA patients.

Characteristic	RA patients (n= 7)
Demographic parameters	
Sex, F/M	7/0
Age, mean (SD), years	50.6 (14.9)
Disease activity	
SJ n, mean (SD)	1.0 (2.2)
TJ n, mean (SD)	6.4 (3.1)
CDAI, mean (SD)	15.9 (5.6)
SDAI, mean (SD)	16.5 (5.8)
DAS28, mean (SD)	4.0 (0.6)
Laboratory values	
ESR, mean (mm/h) (SD)	21.9 (15.5)
CRP, mean (mg/dL) (SD)	6.3 (6.9)
RF positivity, n	3/7
ACPA positivity, n	5/7
Features, n	
Dyslipidemia	1/7
Hypertension	1/7
Diabetes (type 2)	0/7
Pulmonary disease	0/7
Osteoporosis	0/7
Smoking habit	4/7
Therapy, n	
No treatment	3/7
MTX	3/7
Sulfasalazine	1/7

SJ, swollen joints; TJ, tender joints; CDAI, Clinical Disease Activity Index; SDAI, Simple Disease Activity Index; DAS28, Disease ActivityScore on 28 joints; ESR, Erytrocyte Sedimentation Rate; CRP, C-Reactive Protein; RF, Rheumatoid Factor; ACPA, Anti-citrullinated peptide antibodies; MTX, Methotrexate.

### 2.2 Isolation of EMVs

Peripheral blood samples were collected from RA patients and HDs by venipuncture in 5 ml tubes containing sodium citrate as an anticoagulant. To obtain platelet-poor plasma, the samples were centrifuged two times at 2500g for 15 min at room temperature (RT). The platelet-poor plasma was transferred in tubes and filled up to 2 ml with phosphate-buffered saline (PBS) to prevent collapse during the next centrifugation procedure, then they were centrifuged at 14000g for 35 min at 4°C to collect EMVs fraction. The obtained pellets were washed once, at 14000g for 35 min at 4°C, using 2 ml of PBS and subsequently resuspended in 200 μl of PBS (24).

### 2.3 NanoSight analysis

NanoSight NS300 (Malvern Panalytical, Ltd, Malvern, UK) analysis was used to measure size and concentration of EMVs isolated from RA patients and from HDs. To get a suitable concentration, the samples were diluted 1:100 in particle-free PBS (0.02 mm filtered). The instruments were equipped with a 488 nm laser (blue), a high sensitivity sCMOS camera and a syringe pump that flowed the sample at speed of 30 arbitrary units (that indicates the relative speed at which the syringe pump is dispensing the sample). This syringe pump acquires 5 videos, each 60 seconds long under automated script control and analysis was conducted using NTA 3.4 Build 3.4.4 software.

### 2.4 Analysis by western blot

EMVs from all RA patients and HDs were lysed in RIPA buffer (100 mM NaCl, 1 mM EDTA, 1% TRITON X-100, 10 mM Tris-HCl pH 7.4, 0.5% Na-deoxycholate, 0.1% SDS, Na_3_VO_4_) with a protease inhibitor cocktail (Sigma, Milan, Italy). The lysates were centrifuged at 15000g at 4°C for 15 min to obtain soluble proteins. The protein concentration of the EMV lysates was determined using the Bradford assay (Bio-Rad, Segrate, MI, Italy), and lysates were also subjected to 10% SDS-PAGE gel.

The proteins were then transferred onto polyvinylidene difluoride (PVDF) membranes (Bio-Rad).

The membranes were blocked with 5% non-fat dried milk in Tris-buffered saline (TBS) containing 0.05% Tween-20 and then incubated with the listed antibodies: mouse anti-Annexin A1 (Santa Cruz Biotechnology, Dallas, TX, USA), rabbit anti-β-tubulin, rabbit anti-CD63 (Abcam, Cambridge, UK), rabbit anti-CD81 (Abcam), rabbit anti-ALG-2 interacting protein X (ALIX) (Abcam), rabbit anti-citrulline (Millipore, Billerica, MA, USA) or rabbit anti-Carbamyl Lysine (CliniSciences, Nanterre, France) antibodies (Abs), followed by horseradish peroxidase–conjugated anti-rabbit or anti-mouse IgG Abs (Sigma). Immunoreactivity was assessed by the chemiluminescence reaction, using the Clarity Western ECL substrate detection system (Bio-Rad). National Institutes of Health ImageJ 1.62 software, by Mac OS X (Apple Computer International, Cupertino, CA, USA), was employed to perform densitometric scanning analysis.

### 2.5 *In vitro* culture of human monocyte-derived dendritic cells

Peripheral blood mononuclear cells (PBMCs) were isolated from buffy coats obtained from healthy blood donors (HDs), using density gradient centrifugation (Lympholite; Cedarlane, Hornby, Ontario, Canada). PBMCs were incubated with anti-CD14-coated microbeads (Miltenyi Biotec, Gladbach, Germany), and monocytes were sorted with the magnetic device MiniMacs Separation Unit (Miltenyi Biotec), according to the manufacturer’s instructions. Monocyte-derived DCs (termed immature DCs), were obtained by culturing adherent monocytes for 5 days in complete medium [RPMI 1640 supplemented with 1% nonessential amino acids, 1% sodium pyruvate, 10,000 U/ml penicillin-streptomycin (Gibco, Karlsruhe, Germany), 5 × 10^−5^ M 2-mercaptoethanol (Merck, Darmstadt, Germany), and 10% fetal bovine serum (Hyclone Laboratories, Logan, UT, USA)] supplemented with 100 ng/ml recombinant human (rh) Granulocyte-Macrophage Colony-Stimulating Factor (GM-CSF) and 25 ng/ml rh interleukin-4 (IL-4). Trypan blue exclusion assay (Sigma) and light microscope (Nikon Eclipse Ni-U, Nikon Corporation, Tokyo, Japan) were used to assess cell viability and cell morphology, respectively. Immature DCs for (0.8 × 10^6^ cells/ml), were exposed to EMVs (2 x 10^8^/ml) derived from RA patients or HDs at 37°C and 5% CO_2_ for 18 hours. Immature DCs were stimulated with 0.1 μg/ml lipopolysaccharide (LPS, from Escherichia coli strain 0111:B4; Sigma) to obtain control mature DCs. To exclude the possibility of endotoxin contamination in the EMVs, experiments were also conducted in the presence of polymyxin B (10 μg/ml; Sigma).

### 2.6 Flow cytometric analysis of dendritic cell phenotype

Phenotypic surface markers were determined by staining DCs for 30 min at 4°C with a panel of mouse anti-human monoclonal antibodies (mAbs): fluorescein isothiocyanate (FITC) anti-CD14, phycoerythrin (PE) anti-CD1a, Vioblue anti-CD86 (B7-2), PE anti-CD83, Peridinin-Chlorophyll-Protein (PerCP)-Vio700 anti-human leukocyte antigen-D region- related (HLA-DR) and allophycocyanin (APC) anti-CD80 (B7-1), (Miltenyi Biotec). Isotype-control antibodies served as negative controls. To exclude dead cells from analysis, 1 μM Sytox Blue nucleic acid staining (Molecular Probes, Carlsband, CA, USA) was used to exclude cell debris from analysis, and data from 10,000 viable cells were acquired by Gallios Flow cytometer (Beckman Coulter, Brea, CA, USA) and data were analyzed with Kaluza Analysis Software v. 2.1 (Beckman Coulter).

### 2.7 Dendritic cell allostimulatory ability

The allostimulatory ability of both stimulated and unstimulated DCs was assessed using a standard mixed lymphocyte reaction (MLR). CD4+ T cells were isolated from PBMCs through positive selection using anti-CD4 MicroBeads (Miltenyi Biotec). These allogeneic resting T cells (5× 10^5^ cells per well) were labeled with 1 μM carboxyfluorescein succinimidyl ester (CFSE, Thermo Fisher Scientific, Milan, Italy) for 30 minutes. The labeling process was followed by two washes with cold complete medium. Subsequently, the labeled T cells were co-cultured with irradiated DCs at a 1:10 ratio (DCs to T cells) in 24-well plates. T cells collected at the beginning of the co-culture served as the T0 positive control. After 3 days, cells were harvested, and CFSE fluorescence was analyzed by Gallios Flow cytometer (Beckman Coulter). The proliferation index (PI), which measures the number of cell divisions that have occurred, is calculated by using the median fluorescence intensity (MFI) of non-proliferating CFSE^high^ T-cells (peak fluorescence intensity of the viable non-divided cells, MFI nd) and the MFI of all viable CFSE^+^ T-cells (MFI all). The formula for PI=log(MFInd/MFIall)/log2.

### 2.8 Cytokine secretion analysis in dendritic cell culture supernatants

Immature DCs were seeded in 24-well flat-bottom tissue culture plates (Corning, Costar Tewksbury, MA, USA) at a concentration of 0.8 × 10^6^ cells/ml and subsequently stimulated as previously described for 18 hours. The concentrations of IL-12p70, IL-10, and IL-1β in the DC culture supernatants were measured using ELISA (OptEIA kits; BD-Biosciences, San Jose, CA, USA), following the manufacturer’s instructions.

### 2.9 Western blot analysis of MAPK and NF-κB activation

DCs, untreated and treated with EMVs from RA patients (2 x 10^8^/ml), EMVs from HDs (2 x 10^8^/ml) or LPS (0.1 μg/ml) for 15 and 45 min at 37°C and 5% CO_2_ were resuspended in RIPA lysis buffer and the lysates were analyzed by western blot as described above. The PVDF membranes containing the samples stimulated for 15 min were incubated with polyclonal rabbit anti-phospho-ERK1/2 or anti-phospho-p38 Abs (Cell Signaling, Inc. Danvers, MA, USA); alternatively, the PVDF membranes containing samples stimulated for 45 min were incubated with polyclonal rabbit anti-phospho NF-κB-p65 (Cell Signaling, Inc.). Then, both the PVDF membranes were incubated with HRP-conjugated anti-rabbit IgG (Sigma). As loading controls, the phospho-ERK1/2, phospho-p38 and phospho-NF-κB-p65 membranes were stripped and subsequently re-probed respectively with rabbit anti-ERK1/2, anti-p38, anti-NF-κB-p65 (Cell Signaling, Inc) or, alternatively, with anti-β-tubulin antibody or anti β-actin monoclonal Abs (Sigma).

For the reactivity detection, the chemiluminescence reaction using the Clarity Western ECL substrate detection system (Bio-Rad) was employed. Densitometric analysis was conducted using National Institutes of Health ImageJ 1.62 software by Mac OS X (Apple Computer International), allowing the assessment of the absolute value density of each band on the same gel.

### 2.10 Statistical analysis

The Shapiro–Wilk test was applied to test data normality. Normally distributed data were analyzed using an exploratory one-way-ANOVA. If ANOVA showed a significant effect, a Tukey *post hoc* test or independent Student’s t-test was conducted for group comparisons. Values of p <0.05 were considered statistically significant. All the statistical analyses were performed by GraphPad Prism 8.0 software Inc. (San Diego, CA, USA).

## 3 Results

### 3.1 Analysis of EMVs from plasma of naïve RA patients by NanoSight and detection of citrullinated and carbamylated proteins by western blot

**Figure 1:**
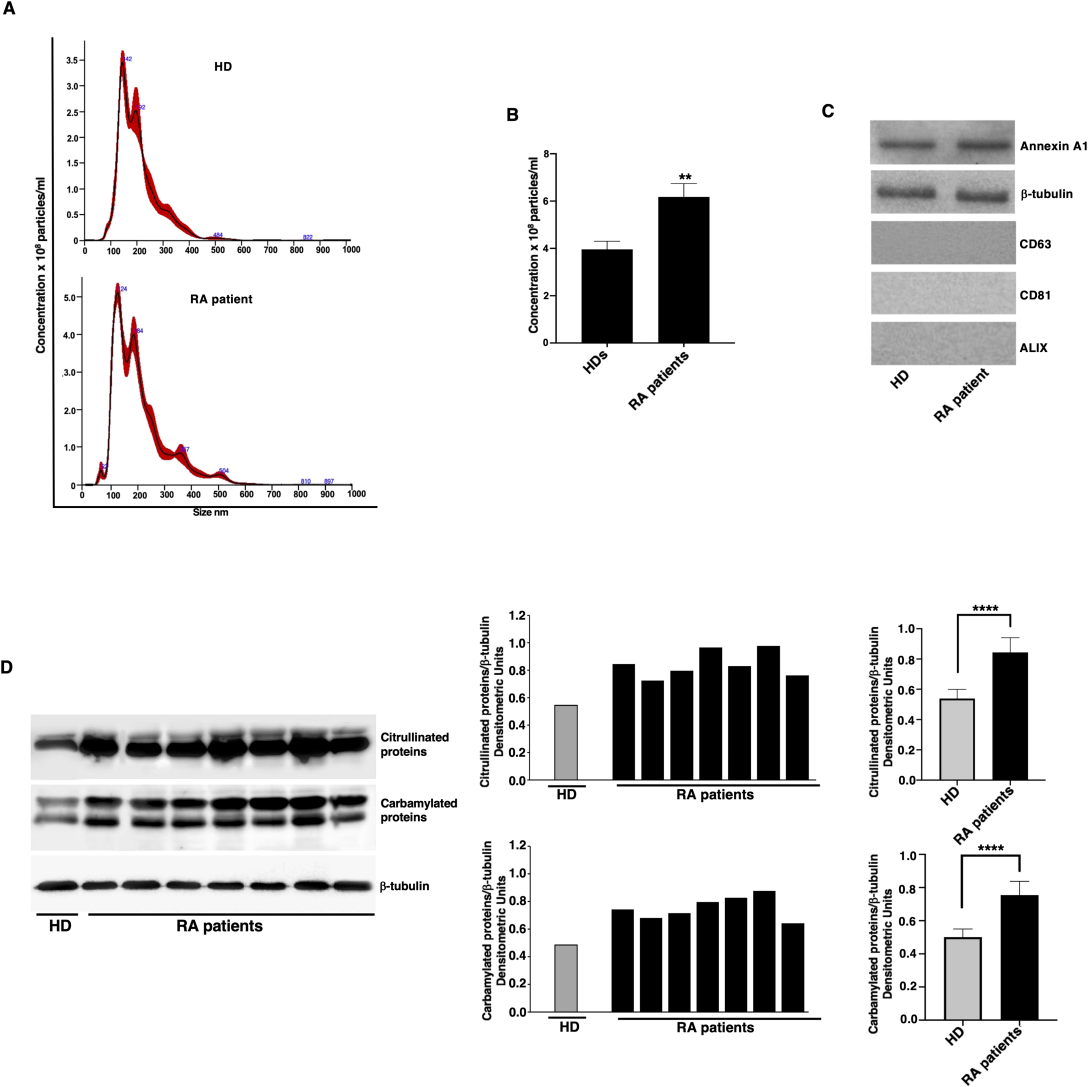
Quantification and analysis of post-translational modification levels in EMVs from RA patients. **(A)** Quantitative analysis of EMVs from one representative RA patient without drug treatment and from one representative HD by NanoSight (Nanoparticle Tracking Analysis). There were five recording videos of 60 s per measurement. **(B)** Quantification of EMVs, the number of EMVs in RA patients was 6.18 x 10^8^ EMVs/ml (S.D. 2.52 x 10^7^), compared to 3.96 x 10^8^ EMVs/ml (S.D. 1.51 x 10^7^) in HDs. **p < 0.01 *vs* HDs. **(C)** Characterization of the EMVs of patients with RA and HD by western blot analysis, using anti-Annexin A1, anti-β-tubulin, anti-CD63, anti-CD81 and anti-ALIX antibodies **(D)** EMVs from RA patients and HDs were analyzed by western blot using anti-citrulline polyclonal or anti-Carbamyl-Lysine polyclonal antibodies. Loading control was evaluated using anti-β-tubulin antibodies. Blots and densitometric analysis of the RA patients and a representative HD are shown. ****p < 0.0001.

The presence of EMVs was preliminary analyzed in the plasma of patients with RA naïve to biological therapy and HDs by NanoSight (Nanoparticle Tracking Analysis), useful for allowing a quantitative analysis. Results showed that the number of EMVs in the RA patients was significantly higher (6.18 x 10^8^ MVs/ml; S.D. 2.52 x 10^7^) compared to those detected in HD plasma (3.96 x 10^8^ MVs/ml; S.D. 1.51 x 10^7^) (**Figures 1A, B**). The purity of EMVs preparation was checked by western blot, which revealed that they express typical markers of EMVs from cell plasma membrane (Annexin A1 and β-tubulin), but not exosomal markers (CD63, CD81, ALIX) (**Figure 1C**).

These findings are in line with previous studies demonstrating an elevated level of circulating EMVs in various inflammatory and autoimmune conditions, suggesting a potential role of EMVs in disease pathogenesis and progression (25, 26).

In parallel experiments, we also analyzed in EMVs the main post-translational modifications of proteins, using anti-citrulline polyclonal Abs or anti-Carbamyl-Lysine polyclonal Abs (**Figure 1D**). As shown, western blot analysis highlighted the appearance of numerous bands, corresponding to citrullinated and carbamylated proteins. Histograms obtained by densitometric analysis revealed that both types of modified proteins were significantly higher in EMVs from RA patients compared to those from HD (**Figure 1D**, right panel).

### 3.2 EMVs from plasma of patients with RA induce phenotypical dendritic cell maturation

**Figure 2:**
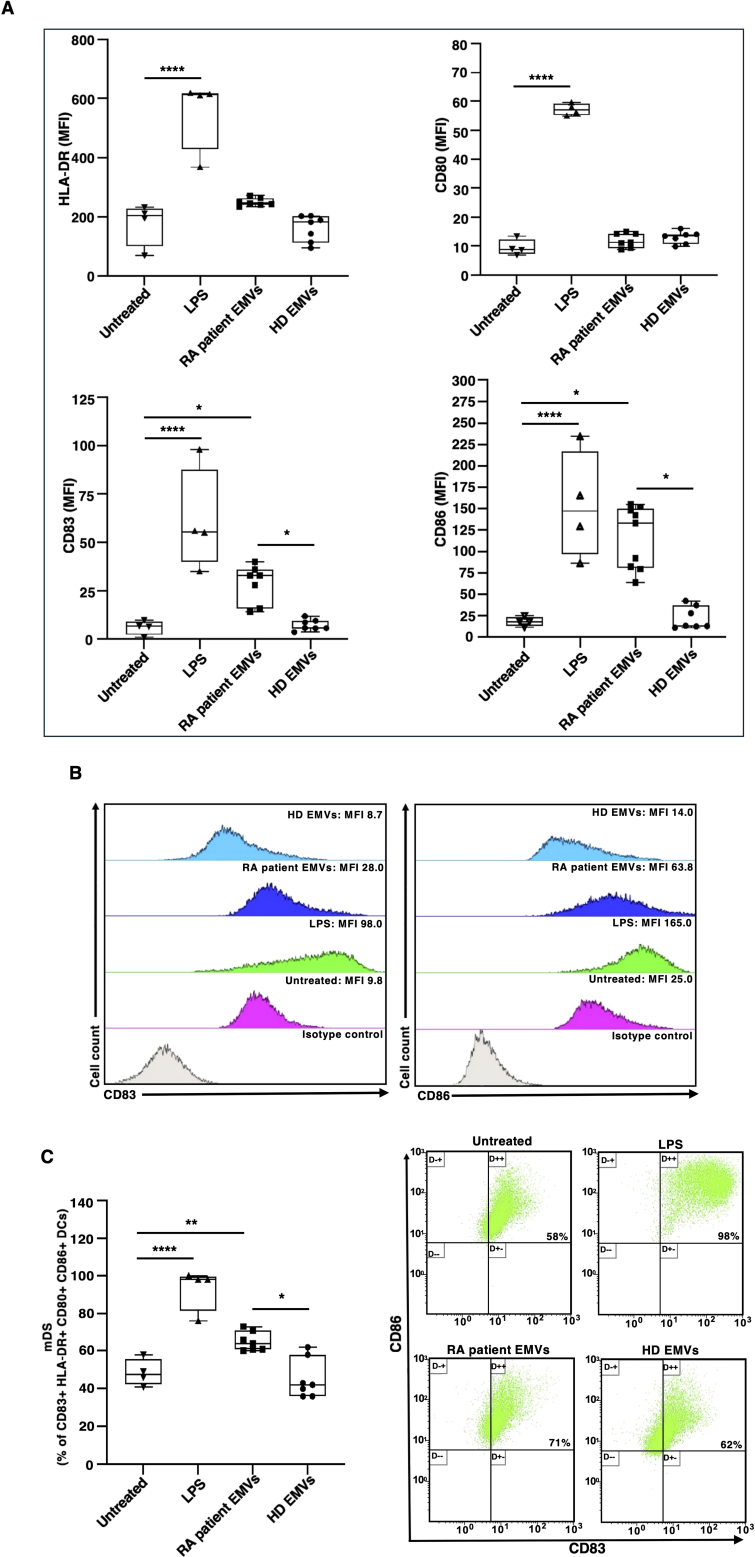
Surface marker expression on immature monocyte-derived dendritic cells (iDCs) after *in vitro* exposure to EMVs from plasma. Immature DCs (8 × 10^5^ cells/ml) were stimulated with or without EMVs from the plasma of patients with RA, HDs, or with LPS and then a four-color flow cytometry analysis was performed to evaluate surface marker expression. **(A)** Histograms show the mean fluorescence intensity (MFI) of HLA-DR, CD80, CD83 and CD86 expression. **(B)** Representative two-dimensional flow cytometry plot images showing the fluorescence intensity of CD83 and CD86 under different conditions. **(C)** Left panel: Histograms represent the mean percentage of positive cells (%) within the DC population. Right panel: Representative flow cytometry dot plots showing the percentage of CD83+ CD86+ DCs within the CD80+ HLA-DR+ DC population. Results are expressed as mean value ± SD of four independent experiments. Significance was determined by one-way ANOVA followed by Tukey’s *post hoc* analysis; *p < 0.05; **p < 0.01****p < 0.0001.

To analyze the change in the characteristics of DCs after EMV treatments, we used flow cytometry to measure the expression of the DC maturation marker CD83, as well as the costimulatory molecules CD86, HLA-DR, and CD80. Untreated DCs exhibited an immature phenotype with low immunoreactivity for CD83, CD86, HLA-DR, and CD80 (represented as mean fluorescence intensity, MFI; see **Figure 2A**). When exposed to the inflammatory stimulus LPS, there was a significant increase in the expression of all maturation markers compared to the untreated control DCs. Furthermore, treatment of immature DCs with EMVs derived from plasma of patients with RA resulted in DC maturation, as evidenced by increased expression of CD83 and CD86 (as MFI; **Figures 2A, B**) respect to both HD EMV treated-DCs and untreated DCs. Treatment with EMVs from RA patients caused an increased percentage of DCs that were positive for the CD83, CD86, HLA-DR and CD80 markers after 18 hours (**Figure 2C**).

### 3.3 EMVs from plasma of patients with RA induce MAPK and NF-κB activation in dendritic cells

**Figure 3:**
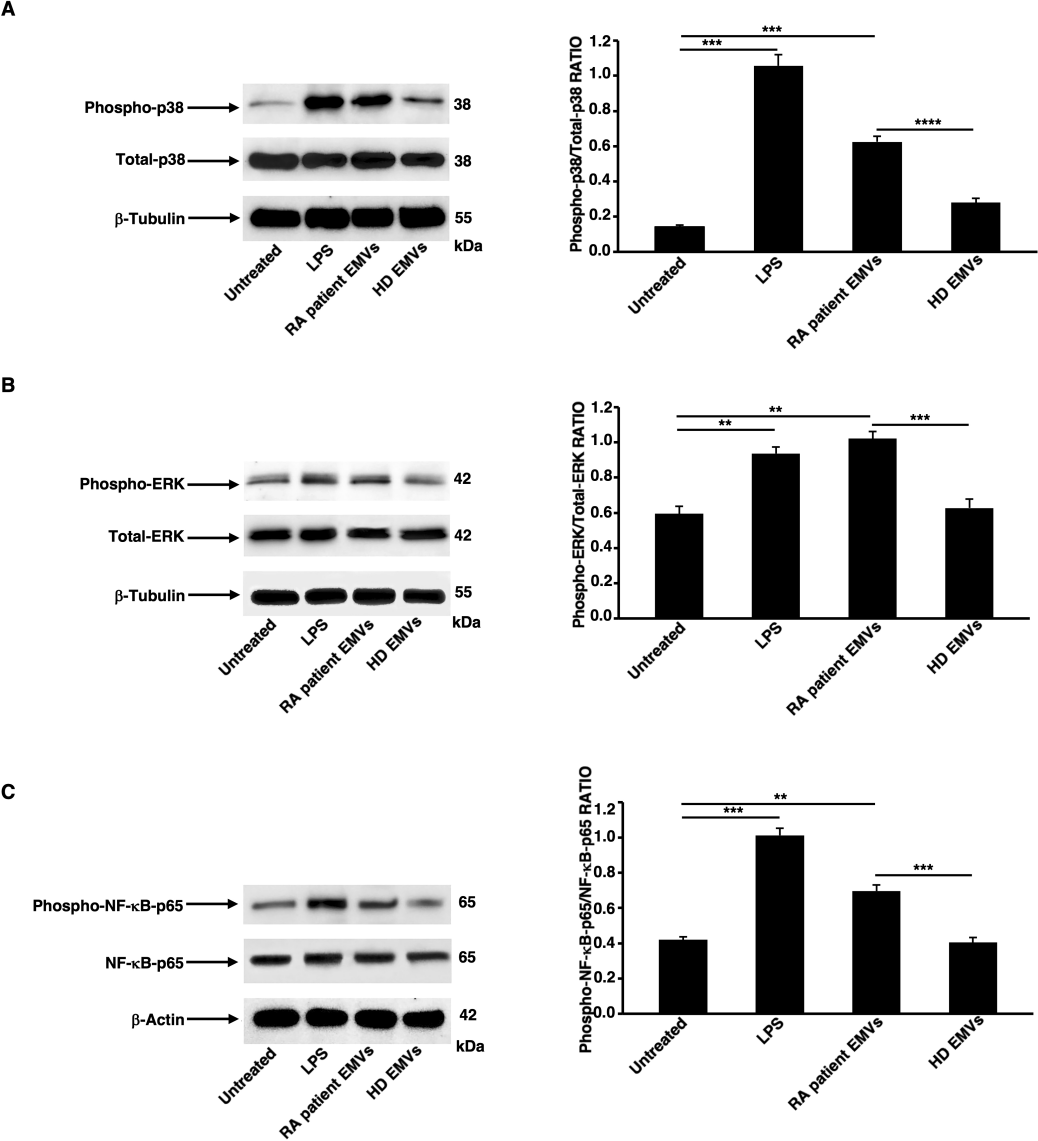
Analysis of MAPK and NF-κB activation in DCs stimulated with RA patient EMVs. DCs untreated or stimulated with LPS, RA patient EMVs and HD EMVs were lysed and analyzed by western blot to evaluate: **(A)** Phospho-p38 expression using rabbit anti-phospho-p38 Ab. **(B)** Phospho-ERK expression using rabbit anti-phospho-ERK1/2 Ab. **(C)** phospho-NF-κB-p65 expression using rabbit anti-phospho-NF-κB-p65 Ab. Anti-β-tubulin mAb or anti-β-actin mAb were used to evaluate loading controls. Samples from one representative RA patient and HD are shown. Densitometric phospho-proteins/total proteins ratios were calculated in all HDs and RA patients and are summarized by histograms in the right panels of the figure. Data are reported as mean (S.D.). Statistical analysis indicated: **p < 0.01; ***p < 0.001; ****p < 0.0001.

We examined the phosphorylation of p38, ERK and NF-κB p65 molecules, to study the effect of RA patient EMVs on signaling that potentially play a role in DC activation. Analysis, by western blot, of cell lysates from DCs showed that RA patient EMVs, as well LPS, induced a significant increase of p38 (**Figure 3A**), ERK (**Figure 3B**) and NF-κB p65 (**Figure 3C**) phosphorylation, compared to both untreated and HD EMVs treated cells. Quantitative analysis confirmed these data (see histograms, right panels, **Figure 3**).

These findings are consistent with the role of these signaling molecules in proinflammatory responses.

### 3.4 EMVs from plasma of patients with RA induce functional mature dendritic cells

**Figure 4:**
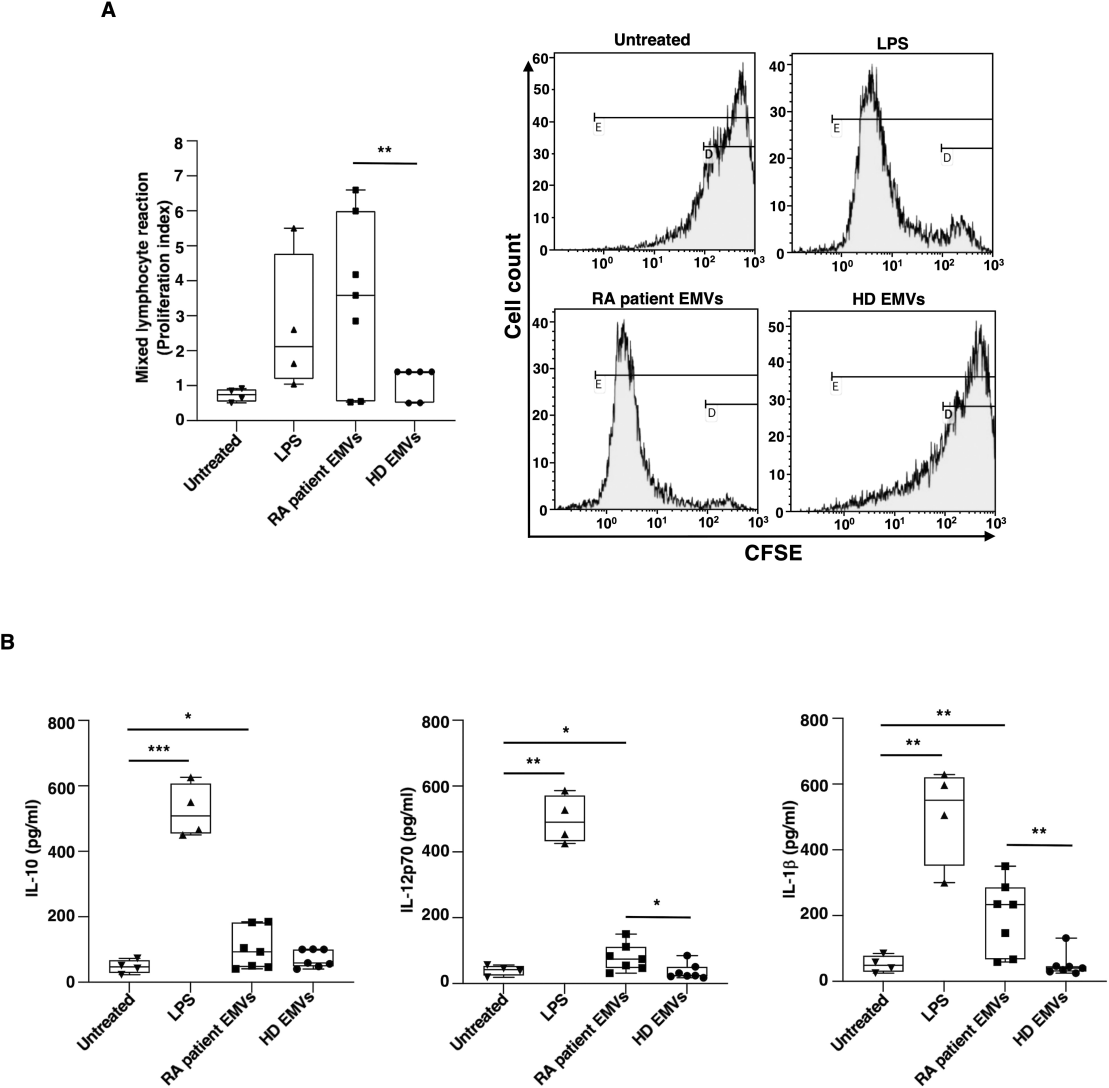
Allostimulatory ability and cytokine production of immature monocyte-derived dendritic cells (iDCs) after *in vitro* exposure to EMVs from plasma. Immature DCs (8 × 10^5^ cells/ml) were stimulated with or without EMVs from the plasma of patients with RA, HDs, or with LPS. **(A)** Left panel: Histogram showing the proliferation index (PI) of CFSE^+^ allogeneic T-cells cultured with iDCs for 3 days at 1:10 DC/T cells analyzed by flow cytometry, as described in Material and methods section; right panel: representative flow cytometry histograms displaying CFSE+ allogeneic T-cell proliferation and calculated PI. Only the percentages of CFSE+ T-cells with lowest CFSE value (CFSE^low^) were counted as proliferating T cells. Results are expressed as mean value ± SD of 4 independent experiments. **(B)** Cytokine production in supernatants collected after 18 h analyzed by specific ELISA experiments in treated or untreated iDC. Significance was determined by one-way ANOVA followed by Tukey’s *post hoc* analysis; *p < 0.05; **p < 0.001.

Given the pivotal role that DCs play in initiating and regulating immune responses, we evaluated the allostimulatory ability in a standard MLR. In these experiments, we provide direct evidence that RA patient EMVs, as well as LPS, enhanced the capacity of DCs to stimulate T-cell proliferation in an allogenic MLR (**Figure 4A**).

Additional details regarding DC activation induced by EMVs from RA patients were obtained through experiments examining the cytokine production in DC supernatants. After 18 h of culture, EMVs isolated from the plasma of RA patients have been shown to stimulate DCs to produce proinflammatory cytokines, specifically IL-12p70 and IL-1β, as well as IL-10 (**Figure 4B**).

These findings support the view that EMVs from RA patients interact and activate DCs thus inducing a proinflammatory mature phenotype.

## 4 Discussion

Our *in vitro* study provides new insights showing the interaction between EMVs from plasma of RA patients and DCs. Our findings strongly suggest that the increase of PTMs of proteins in EMVs of RA patients is an event that makes these vesicles able to activate immature monocyte-derived DCs from HDs (27–30).

We are preliminary confirmed that carbamylated and citrullinated proteins are present in EMVs from the patients with RA, according to our previous observations (19). These findings are consistent with the known role of PTMs in RA pathogenesis. The higher levels of PTM proteins in EMVs from RA patients suggest their potential contribution to the autoimmune response in RA by promoting the presentation of modified autoantigens and perpetuating inflammatory signaling pathways (19, 31).

Moreover, the main finding of this study was the observation that EMVs from plasma of patients with RA interact and activate DCs, thus inducing a proinflammatory phenotype. Surface marker phenotyping indicated that EMV treated-DCs exhibit increased expression of maturation marker CD83 and costimulatory molecule CD86, as well as a higher percentage of CD83+ HLA-DR+ CD80+ CD86+ cells. The increased expression of CD83 and CD86 indicates that the DCs are in a mature state (28, 32, 33) ready to effectively present antigens and provide necessary signals for T cell activation and differentiation. Moreover, the increased percentage of DCs expressing costimulatory markers suggests a broad enhancement of their antigen-presenting capabilities and a predisposition to initiate adaptive immune responses. Our findings are consistent with the prior observation indicating the proinflammatory function of circulating exosomes from systemic lupus erythematosus patients on peripheral mononuclear cells (34), as well as their ability to activate DC subsets and prime neutrophils for NETosis (35).

Thus, in the present study, our experiments provided significant insights into the activation of dendritic cells induced by EMVs from the plasma of patients with RA. The data showed that these EMVs significantly enhanced the allostimulatory ability of DCs. This finding is particularly relevant, as it demonstrates that EMVs can amplify the capacity of DCs to stimulate T-cell proliferation in an allogeneic MLR.

This enhancement of DC allostimulatory function leads to considering that EMVs act as powerful danger signals, which can activate DCs. The activation of DCs is a critical step in the immune response, leading to the maturation of these cells and the subsequent presentation of antigens to T cells. In the context of RA, in which the immune system is dysregulated, the ability of EMVs to further stimulate DC activation could contribute to the pathogenesis of the disease. Our findings support that EMVs from RA patients include components that can trigger DC activation, responsible for an enhanced immune response. This could potentially explain the chronic inflammation observed in RA, as continuously activated DCs would perpetuate the activation and proliferation of autoreactive T cells.

Data on DC activation were strongly supported by biochemical data, which demonstrated that EMVs from plasma of RA patients induce MAPK and NF-κB activation in DCs. It is known that the p38 MAPK pathway is involved in the production of proinflammatory cytokines and the activation of inflammatory cells (36), while ERK signaling has been implicated in the maturation and function of DCs, influencing their ability to present antigens and activate T cells (37). In addition, NF-κB is a key regulator of immune and inflammatory responses, controlling the expression of genes involved in cytokine production, cell survival, and proliferation (38). Previous studies have demonstrated that EVs from various sources can modulate these signaling pathways. For example, tumor-derived exosomes have been shown to activate NF-κB signaling in DCs, promoting a proinflammatory phenotype (39). Similarly, EVs from viral infections can trigger p38 and ERK pathways, leading to enhanced immune responses (40). The significant increase in the phosphorylation of p38, ERK and NF-κB p65, observed after the treatment with RA patient EMVs, suggests that these vesicles carry bioactive molecules capable of potent immune activation, potentially contributing to the chronic inflammation observed in RA (41).

Moreover, EMVs isolated from the plasma of RA patients have been shown to stimulate DCs to produce IL-12p70 and IL-1β, inducing a proinflammatory phenotype in DCs. This observation is supported by previous studies indicating that EMVs play a crucial role in immune modulation and inflammation (41, 42). While cytokine production was also observed for EMVs isolated from healthy individuals, the levels were significantly lower compared to those from RA patients. This is consistent with findings that EMVs from diseased states often have altered cargo and enhanced bioactivity (43). Furthermore, the production of proinflammatory cytokines by DCs was also observed following stimulation with LPS, a powerful activator of the innate immune response. This supports the hypothesis that EMVs from RA patients carry unique signaling molecules that enhance the inflammatory response (44). Therefore, the EMVs from RA patients induced a proinflammatory phenotype in DCs, suggesting a potential mechanism by which RA microenvironment perpetuates inflammation through the modulation of DC function. These findings highlight the importance of EMVs in the pathogenesis of RA and their potential as therapeutic targets (45, 46).

A limitation of our study is that we assessed the effects of EMVs exclusively in monocyte-derived dendritic cells. However, diverse DC subsets, such as myeloid DCs (mDCs) and plasmacytoid DCs (pDCs), are implicated in RA (47–49). Each subset has unique characteristics and functions that contribute to the inflammatory milieu in RA. Future research should investigate both mDCs and pDCs to determine if the enhanced proinflammatory phenotype induced by EMVs from RA is consistent across different DC subsets. These results highlight the potential of targeting EMVs or their signaling pathways as a strategy to control DC activation and mitigate the aberrant immune responses of RA. Future research should focus on identifying the molecular mechanisms through which EMVs exert their effects on DCs and exploring how these pathways can be manipulated to develop novel treatments for RA. Indeed, understanding the intricate interplay between DCs, post-translational modifications, and microvesicles in the context of RA pathogenesis is crucial for developing targeted therapies and improving patient outcomes. This knowledge can lead to the identification of novel therapeutic targets and biomarkers, which may help in early diagnosis, predicting disease progression, and tailoring personalized treatment strategies. Furthermore, it can provide deeper insights into the underlying mechanisms of RA, potentially revealing new avenues for preventing or mitigating the impact of the disease. Through continued research in this area, we can improve our ability to manage RA more effectively and consequently the quality of life of patients suffering from this disabling condition (5, 20, 50).

## Data Availability

The raw data supporting the conclusions of this article will be made available by the authors, without undue reservation.

## References

[1] Smolen JS Aletaha D McInnes IB. Rheumatoid arthritis. Lancet. (2016) 388:2023–38. doi: 10.1016/S0140-6736(16)30173-8 27156434

[2] Radu AF Bungau SG. Management of rheumatoid arthritis: an overview. Cells. (2021) 10:2857. doi: 10.3390/cells10112857 34831081 PMC8616326

[3] Gravallese EM Firestein GS. Rheumatoid arthritis—Common origins, divergent mechanisms. N Engl J Med. (2023) 388:529–42. doi: 10.1056/NEJMra2103726 36780677

[4] Jang S Kwon EJ Lee JJ. Rheumatoid arthritis: pathogenic roles of diverse immune cells. Int J Mol Sci. (2022) 23:905. doi: 10.3390/ijms23020905 35055087 PMC8780115

[5] Suwa Y Nagafuchi Y Yamada S Fujio K. The role of dendritic cells and their immunometabolism in rheumatoid arthritis. Front Immunol. (2023) 14:1161148. doi: 10.3389/fimmu.2023.1161148 37251399 PMC10213288

[6] Edilova MI Akram A Abdul-Sater AA. Innate immunity drives pathogenesis of rheumatoid arthritis. BioMed J. (2021) 44:172–82. doi: 10.1016/j.bj.2020.06.010 PMC8178572 32798211

[7] Ioan-Facsinay A el-Bannoudi H Scherer HU van der Woude D Ménard HA Lora M. Anti-cyclic citrullinated peptide antibodies are a collection of anti-citrullinated protein antibodies and contain overlapping and non-overlapping reactivities. Ann Rheum Dis. (2011) 70:188–93. doi: 10.1136/ard.2010.131102 20736390

[8] Shi J van Veelen PA Mahler M Vanholder R Kalim S Jankowski J. Carbamylation and antibodies against carbamylated proteins in autoimmunity and other pathologies. Autoimmun Rev. (2014) 13:225–30. doi: 10.1016/j.autrev.2013.10.008 24176675

[9] Pruijn GJ. Citrullination and carbamylation in the pathophysiology of rheumatoid arthritis. Front Immunol. (2015) 6:192. doi: 10.3389/fimmu.2015.00192 25964785 PMC4410602

[10] Noels H Jankowski V Schunk SJ Vanholder R Kalim S Jankowski J. Post-translational modifications in kidney diseases and associated cardiovascular risk. Nat Rev Nephrol. (2024) 20:495–512. doi: 10.1038/s41581-024-00837-x 38664592

[11] Zhong Q Xiao X Qiu Y. Protein posttranslational modifications in health and diseases: Functions, regulatory mechanisms, and therapeutic implications. Med Commun. (2020) 4:e261. doi: 10.1002/mco2.261 PMC10152985 37143582

[12] Trouw LA Rispens T Toes REM. Beyond citrullination: other post-translational protein modifications in rheumatoid arthritis. Nat Rev Rheumatol. (2017) 13:331–9. doi: 10.1038/nrrheum.2017.15 28275265

[13] Sorice M Iannuccelli C Manganelli V Capozzi A Alessandri C Lococo E. Autophagy generates citrullinated peptides in human synoviocytes: a possible trigger for anti-citrullinated peptide antibodies. Rheumatol (Oxford). (2016) 55:1374–85. doi: 10.1093/rheumatology/kew178 27074807

[14] Manganelli V Recalchi S Capozzi A Riitano G Mattei V Longo A. Autophagy induces protein carbamylation in fibroblast-like synoviocytes from patients with rheumatoid arthritis. Rheumatol (Oxford). (2018) 57:2032–41. doi: 10.1093/rheumatology/key174 29982776

[15] Spinelli FR Pecani A Conti F Mancini R Alessandri C Valesini G. Post-translational modifications in rheumatoid arthritis and atherosclerosis: Focus on citrullination and carbamylation. J Int Med Res. (2016) 44:81–4. doi: 10.1177/0300060515593258 PMC5536531 27683146

[16] Mondal S Thompson PR. Chemical biology of protein citrullination by the protein A arginine deiminases. Curr Opin Chem Biol. (2021) 63:19–27. doi: 10.1016/j.cbpa.2021.01.010 33676233 PMC8384633

[17] Wang Z Nicholls SJ Rodriguez ER Kummu O Hörkkö S Barnard J. Protein carbamylation links inflammation, smoking, uremia and atherogenesis. Nat Med. (2007) 13:1176–84. doi: 10.1038/nm1637 17828273

[18] Pecani A Alessandri C Spinelli FR Priori R Riccieri V Di Franco M. Prevalence, sensitivity and specificity of antibodies against carbamylated proteins in a monocentric cohort of patients with rheumatoid arthritis and other autoimmune rheumatic diseases. Arthritis Res Ther. (2016) 18:276. doi: 10.1186/s13075-016-1173-0 27887639 PMC5124308

[19] Ucci FM Recalchi S Barbati C Manganelli V Capozzi A Riitano G. Citrullinated and carbamylated proteins in extracellular microvesicles from plasma of patients with rheumatoid arthritis. Rheumatol (Oxford). (2023) 62:2312–9. doi: 10.1093/rheumatology/keac598 36255236

[20] Riitano G Recalchi S Capozzi A Manganelli V Misasi R Garofalo T. The role of autophagy as a trigger of post-translational modifications of proteins and extracellular vesicles in the pathogenesis of rheumatoid arthritis. Int J Mol Sci. (2023) 24:12764. doi: 10.3390/ijms241612764 37628944 PMC10454292

[21] Liao HJ Hsu PN. Immunomodulatory effects of extracellular vesicles from mesenchymal stromal cells: Implication for therapeutic approach in autoimmune diseases. Kaohsiung J Med Sci. (2024) 40:520–9. doi: 10.1002/kjm2.12841 PMC11895663 38712483

[22] Nicolao MC Rodriguez Rodrigues C Cumino AC. Extracellular vesicles from Echinococcus granulosus larval stage: Isolation, characterization and uptake by dendritic cells. PloS Negl Trop Dis. (2019) 13:e0007032. doi: 10.1371/journal.pntd.0007032 30615613 PMC6344059

[23] Kay J Upchurch KS. ACR/EULAR 2010 rheumatoid arthritis classification criteria. Rheumatol (Oxford). (2012) 51 Suppl 6:vi5–9. doi: 10.1093/rheumatology/kes279 23221588

[24] Barbati C Vomero M Colasanti T Diociaiuti M Ceccarelli F Ferrigno S. TNFα expressed on the surface of microparticles modulates endothelial cell fate in rheumatoid arthritis. Arthritis Res Ther. (2018) 20:273. doi: 10.1186/s13075-018-1768-8 30526655 PMC6286582

[25] Barteneva NS Fasler-Kan E Bernimoulin M Stern JN Ponomarev ED Duckett L. Circulating microparticles: square the circle. BMC Cell Biol. (2013) 22:23. doi: 10.1186/1471-2121-14-23 PMC3651414 23607880

[26] Pisetsky DS Ullal AJ Gauley J Ning TC. Microparticles as mediators and biomarkers of rheumatic disease. Rheumatol (Oxford). (2012) 51:1737–46. doi: 10.1093/rheumatology/kes028 PMC3448881 22403183

[27] Buttari B Profumo E Mattei V Siracusano A Ortona E Margutti P. Oxidized beta2-glycoprotein I induces human dendritic cell maturation and promotes a T helper type 1 response. Blood. (2005) 106:3880–7. doi: 10.1182/blood-2005-03-1201 16099886

[28] Buttari B Profumo E Capozzi A Facchiano F Saso L Sorice M. Advanced glycation end products of human β_2_ glycoprotein I modulate the maturation and function of DCs. Blood. (2011) 117:6152–61. doi: 10.1182/blood-2010-12-325514 21498672

[29] Capozzi A Truglia S Buttari B Recalchi S Riitano G Manganelli V. Carbamylation of β2-glycoprotein I generates new autoantigens for antiphospholipid syndrome: a new tool for diagnosis of ‘seronegative’ patients. Rheumatol (Oxford). (2022) 61:4187–97. doi: 10.1093/rheumatology/keac045 35108369

[30] Buttari B Profumo E Capozzi A Saso L Sorice M Riganò R. Post-translational modifications of proteins in antiphospholipid antibody syndrome. Crit Rev Clin Lab Sci. (2019) 56:511–25. doi: 10.1080/10408363.2019.1650714 31448653

[31] Manfredi M Williams E Cho WC Falasca M. Editorial: recent advances in *in vitro* and *in vivo* multi-omics analyses of extracellular vesicles: therapeutic targets and biomarkers. Front Mol Biosci. (2021) 8:784436. doi: 10.3389/fmolb.2021.784436 34778382 PMC8578700

[32] Cao W Lee SH Lu J. CD83 is preformed inside monocytes, macrophages and dendritic cells, but it is only stably expressed on activated dendritic cells. Biochem J. (2005) 385:85–93. doi: 10.1042/BJ20040741 15320871 PMC1134676

[33] Banchereau J Briere F Caux C Davoust J Lebecque S Liu YJ. Immunobiology of dendritic cells. Annu Rev Immunol. (2000) 18:767–811. doi: 10.1146/annurev.immunol.18.1.767 10837075

[34] Lee JY Park JK Lee EY Lee EB Song YW. Circulating exosomes from patients with systemic lupus erythematosus induce a proinflammatory immune response. Arthritis Res Ther. (2020) 22:109. doi: 10.1186/s13075-016-1159-y 32384933 PMC7210673

[35] Dieker J Tel J Pieterse E Thielen A Rother N Bakker M. Circulating apoptotic microparticles in systemic lupus erythematosus patients drive the activation of dendritic cell subsets and prime neutrophils for NETosis. Arthritis Rheumatol. (2016) 68:462–72. doi: 10.1002/art.39417 26360137

[36] Cuenda A Rousseau S. p38 MAP-kinases pathway regulation, function and role in human diseases. Biochim Biophys Acta. (2007) 1773:1358–75. doi: 10.1016/j.bbamcr.2007.03.010 17481747

[37] Neves BM Cruz MT Francisco V Garcia-Rodriguez C Silvestre R Cordeiro-da-Silva A. Differential roles of PI3-Kinase, MAPKs and NF-kappaB on the manipulation of dendritic cell T(h)1/T(h)2 cytokine/chemokine polarizing profile. Mol Immunol. (2009) 46:2481–92. doi: 10.1016/j.molimm.2009.05.021 19520433

[38] Hayden MS Ghosh S. NF-κB, the first quarter-century: remarkable progress and outstanding questions. Genes Dev. (2012) 26:203–34. doi: 10.1101/gad.183434.111 PMC3278889 22302935

[39] Chow A Zhou W Liu L Fong MY Champer J Van Haute D. Macrophage immunomodulation by breast cancer-derived exosomes requires Toll-like receptor 2-mediated activation of NF-κB. Sci Rep. (2014) 4:5750. doi: 10.1038/srep05750 25034888 PMC4102923

[40] Urbanelli L Buratta S Tancini B Sagini K Delo F Porcellati S. The role of extracellular vesicles in viral infection and transmission. Vaccines (Basel). (2019) 7:102. doi: 10.3390/vaccines7030102 31466253 PMC6789493

[41] György B Szabó TG Pásztói M Pál Z Misják P Aradi B. Membrane vesicles, current state-of-the-art: emerging role of extracellular vesicles. Cell Mol Life Sci. (2011) 68:2667–88. doi: 10.1007/s00018-011-0689-3 PMC3142546 21560073

[42] Yáñez-Mó M Siljander PR Andreu Z Zavec AB Borràs FE Buzas EI. Biological properties of extracellular vesicles and their physiological functions. J Extracell Vesicles. (2015) 4:27066. doi: 10.3402/jev.v4.27066 25979354 PMC4433489

[43] Mathivanan S Ji H Simpson RJ. Exosomes: extracellular organelles important in intercellular communication. J Proteomics. (2010) 73:1907–20. doi: 10.1016/j.jprot.2010.06.006 20601276

[44] Stojanovic A Veselinovic M Zong Y Jakovljevic V Pruner I Antovic A. Increased expression of extracellular vesicles is associated with the procoagulant state in patients with established rheumatoid arthritis. Front Immunol. (2021) 12:718845. doi: 10.3389/fimmu.2021.718845 34394126 PMC8358654

[45] Zhang B Zhao M Lu Q. Extracellular vesicles in rheumatoid arthritis and systemic lupus erythematosus: functions and applications. Front Immunol. (2021) 11:575712. doi: 10.3389/fimmu.2020.575712 33519800 PMC7841259

[46] Robbins PD Morelli AE. Regulation of immune responses by extracellular vesicles. Nat Rev Immunol. (2014) 14:195–208. doi: 10.1038/nri3622 24566916 PMC4350779

[47] Yang GX Lian ZX Kikuchi K Liu YJ Ansari AA Ikehara S. CD4- plasmacytoid dendritic cells (pDCs) migrate in lymph nodes by CpG inoculation and represent a potent functional subset of pDCs. J Immunol. (2005) 174:3197–203. doi: 10.4049/jimmunol.174.6.3197 15749849

[48] Takakubo Y Takagi M Maeda K Tamaki Y Sasaki A Asano T. Distribution of myeloid dendritic cells and plasmacytoid dendritic cells in the synovial tissues of rheumatoid arthritis. J Rheumatol. (2008) 35:1919–31. 18785315

[49] Laranjeira P Pedrosa M Duarte C Pedreiro S Antunes B Ribeiro T. Human bone marrow mesenchymal stromal/stem cells regulate the proinflammatory response of monocytes and myeloid dendritic cells from patients with rheumatoid arthritis. Pharmaceutics. (2022) 14:404. doi: 10.3390/pharmaceutics14020404 35214136 PMC8880255

[50] Thomas R. Dendritic cells and the promise of antigen-specific therapy in rheumatoid arthritis. Arthritis Res Ther. (2013) 15:204. doi: 10.1186/ar4130 23374912 PMC3672739

